# Magnitude processing in non-symbolic stimuli

**DOI:** 10.3389/fpsyg.2013.00375

**Published:** 2013-06-25

**Authors:** Tali Leibovich, Avishai Henik

**Affiliations:** ^1^The Cognitive Neuropsychology Laboratory, Department of Cognitive Sciences, Ben-Gurion University of the NegevBeer-Sheva, Israel; ^2^The Cognitive Neuropsychology Laboratory, Department of Psychology and the Zlotowski Center for Neuroscience, Ben-Gurion University of the NegevBeer-Sheva, Israel

**Keywords:** numerical cognition, ANS, number sense, non-symbolic stimuli, visual properties, discrete, continuous, magnitude processing

## Abstract

Dot arrays are often used to study basic numerical skills across cultures, species and development. Researchers investigate the ability of subjects to discriminate between dot arrays, as a function of the ratio or distance between their numerosities. Such studies have contributed significantly to the number sense theory (i.e., that humans are born with the ability to process numerosities, and share this ability with various species)—possibly the most influential theory in numerical cognition literature today. However, a dot array contains, in addition to numerosity, continuous properties such as the total surface area of the dots, their density, etc. These properties are highly correlated with numerosity and therefore might influence participants' performance. Different ways in which different studies choose to deal with this confound sometimes lead to contradicting results, and in our opinion, do not completely eliminate the confound. In this work, we review these studies and suggest several possible reasons for the contradictions in the literature. We also suggest that studying continuous properties, instead of just trying to control them, may contribute to unraveling the building blocks of numerical abilities.

## Introduction

Humans and animals share the ability to estimate and discriminate magnitudes. In animals, these abilities are crucial for survival. For example, swimming with the larger shoal increases the probability of fish to survive (Agrillo et al., 2008), and estimating the number of lions in the opponent group affects the group's decision to “fight or flight” (McComb et al., 1994). Humans are able to estimate and compare magnitudes from a very early age (Piazza, 2010) and use these abilities every day. It is suggested that our mathematical abilities are built upon these basic skills (Dehaene, 1997; Cantlon et al., 2009; Piazza, 2010).

To study such basic and universal abilities, many researchers use dot arrays to investigate non-symbolic representation of quantities. Discriminating between such arrays is not culture-related and does not require language or formal education, making this type of stimuli ideal for developmental, cross-cultural and animal studies. In these studies, researchers manipulate the numerosity of the dots in the arrays and then ask subjects to compare the numerosities, indicate if two arrays have the same or different numerosity, or indicate which array contains more dots. It is important to remember that dot arrays contain, in addition to numerosity, other visual properties, such as the total surface of the dots, their density, the total space they occupy, etc. We will refer to these properties as continuous properties. Unlike discrete properties (i.e., the number of dots), one can only estimate continuous properties, even without time limitation. For example, no matter how much time you have, you cannot tell the exact cumulative area of the dots in an array. You can only estimate it. This review will deal mainly with visually presented non-symbolic quantities.

Studies involving dot arrays contributed significantly to highly influential theories such as the number sense (Dehaene, 1997) and the approximate number system (ANS) (Cantlon et al., 2009). Dehaene's number sense theory (1997) suggests that both humans and animals are born with a sense for *numbers*. A “number processor” component allows both humans and animals to “represent quantities and transform them according to the rules of arithmetic” (p. 18). The ANS theory (Cantlon et al., 2009) suggests that numerical symbols “build on the approximate number system (ANS) which represents the *number of discrete* objects or events as a continuous mental magnitude” (p. 83). Note that the majority of studies involving dot arrays focus on numerosities; the numerosity is systematically manipulated and the participants' attention is directed to this dimension. Furthermore, as mentioned in a review by Mix et al. (2002), some researchers even suggested that “once numerical competence has been shown, all subsequent development must be guided by discrete number bias” (p. 278). In the current work, we raise two issues—theoretical and methodological—suggesting that numerosity might not be the only visual property that is extracted from a set and is the basis for numerical abilities.

## Theoretical issues

**Figure 1:**
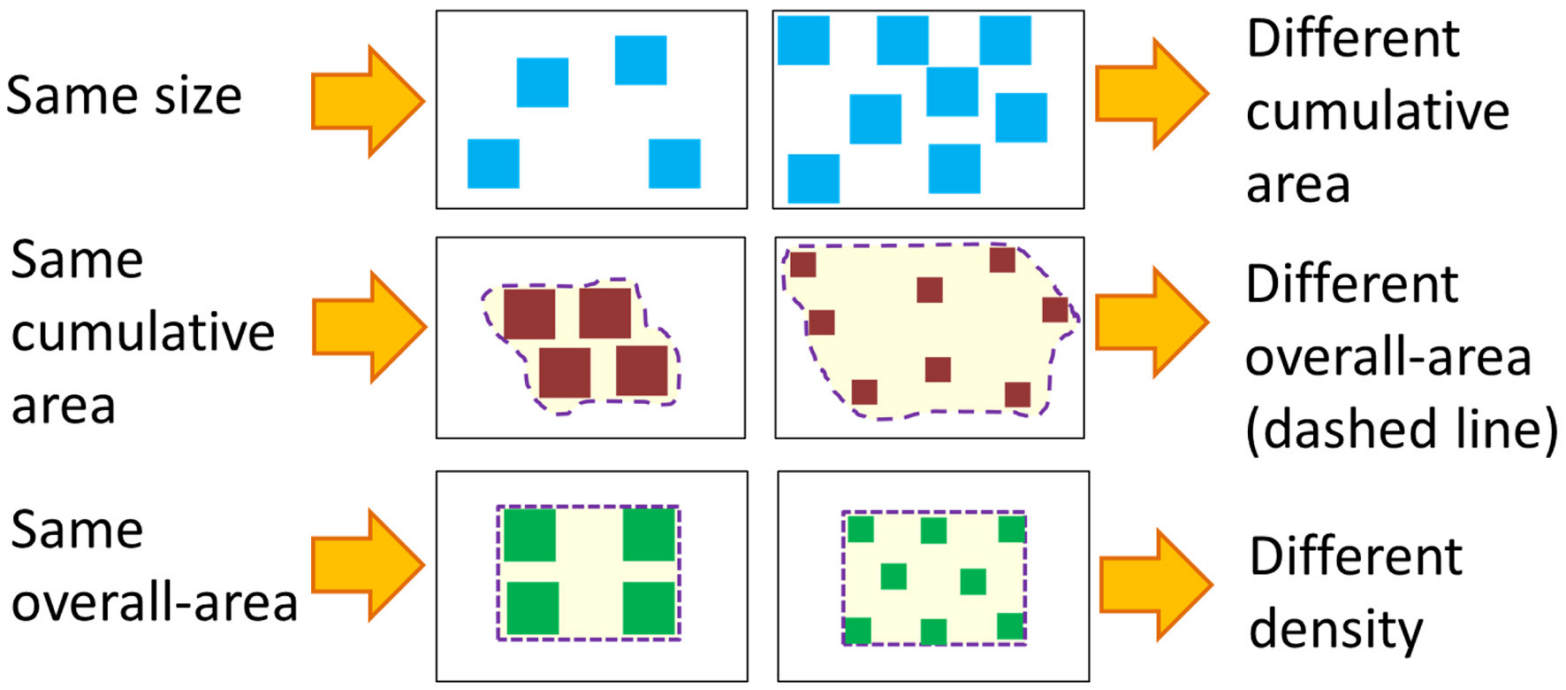
**Illustration—Controlling continuous magnitudes.** Controlling for one continuous magnitude always changes other continuous magnitudes.

There are two ecological factors that must be considered when discussing the influence of discrete and continuous properties on numerical cognition. First, the only non-symbolic way to visually convey numerosities is through arrays of items. These arrays always carry continuous properties that highly correlate with numerosities. To study numerosity in isolation, one must neutralize the effect of all continuous properties at once; namely, the only difference between two arrays (e.g., an array containing four dots and an array containing 10 dots) should be the numerosity, and continuous properties (e.g., the total surface area of the dots, their density, average size, etc.) should be identical. This is physically impossible. For example, if there are four squares in one array and 2 in another array and all the squares are of the same size, the four squares have greater total surface area than the two squares. To control for area, one can equalize the total surface of the two arrays. However, this means that the density and average size will have changed (see Figure 1). Given these high correlations, it seems more plausible to postulate a system that takes into consideration both discrete and continuous properties.

Second, from an evolutionary perspective, in most cases *discrimination* between magnitudes (as opposed to enumeration) is enough to make a decision. For example, fish select a shoal by comparing the sizes of two shoals. It is impossible to determine that their choice was based on the absolute number of fish in a shoal. In contrast, using numerosities might help when one needs to produce an exact value. For animals, this is rarely the case. If animals need to enumerate, it is usually in the subitizing range (i.e., 1–4 items) (Gross et al., 2009). Hence, it is hard to imagine a system that was designed to ignore most visual cues (i.e., continuous properties) and attend only to a discrete one (see also Gebuis and Reynvoet, 2012a,b). For humans, *the need for enumeration is cultural, not innate*. Premack and Premack (2005) argued that cultural changes (the adaptation to new technologies) required the development of exact representation. Studies on hunter-gatherer groups (Gordon, 2004; Pica et al., 2004) are examples of cultures that do not require exact representation of magnitudes. Even the few number words in their lexicon are not comparable to Western number words because they represent *approximate numbers*. Importantly, in the study of Dehaene et al. (2008), 22% of the uneducated Amazonian adults failed to map numbers 1–3 in the right order and 37% of the participants exhibited bimodal mapping, using only the end points of the line (Núñez, 2011).

To summarize, it looks as though the ability to enumerate is cultural and not evolutionary. To survive, estimation and discrimination are enough. The question is *what properties do we use to make that estimation*? We argue here that since it is physically impossible to study discrete magnitudes in isolation, it is difficult to accept theories such as the ANS and the number senses, which suggest numerosity is the most salient cue. It is more likely that we integrate multiple visual cues to make size estimations (Gebuis and Reynvoet, 2012a,b). Another theory that might lead to a similar conclusion (i.e., using all available cues) is the theory of magnitudes—ATOM (Walsh, 2003). The ATOM suggests that all magnitudes, in all modalities, are being represented in the same region in the brain—the parietal lobe—since they are all needed to allow us to physically interact with the environment. In other words, the purpose of all magnitude processing is to guide motor actions. Following this logic, it is more instrumental to use all available cues in the environment—not only discrete ones—to make size estimations.

## Methodological issues

Since neutralizing changes in continuous properties is impossible, different studies chose different ways to control those properties: manipulating only one continuous property at a time (Mussolin and Mejias, 2010—developmental study); assigning a random dot size to each array (Piazza, 2010); using a single array containing two different colors of dots where participants (children and adults) must indicate the color of the more numerous dots (Halberda et al., 2008); etc. Studies using such methods to control for continuous properties share a hidden assumption that participants rely only on one visual cue throughout the entire experiment. Thus, by making this cue an unreliable predictor of numerosity, participants have no choice but to rely on numerosities. It is important to note that different methodologies (imaging, behavioral, computer modeling) used the same logic and similar stimuli. We chose to focus mainly on behavioral studies, although these methodological issues apply to other methodologies as well.

Different means of controlling continuous properties led to contradicting results in the literature. Stoianov and Zorzi (2012) demonstrated the existence of “numerosity detectors” in a computer modeling methodology. However, these detectors received input from a layer that encoded cumulative area. Hurewitz et al. (2006) had adults compare two dot arrays that differed in their numerosity and continuous properties. The numerosity was either congruent or incongruent with surface area. The authors reported that adults automatically processed the continuous properties of the arrays when asked to compare numerosities. They also found the reverse influence (i.e., that numerosity was processed automatically when comparing areas) but to a lesser extent. In response to those results, Barth (2008) suggested that the ratio between the surface areas of the arrays was always greater for incongruent trials, and that the difference between incongruent and congruent trials is actually a ratio effect (i.e., the surface areas of the two arrays were more similar in incongruent than in the congruent condition, resulting in longer reaction times for incongruent trials), and not an interference effect. Nys and Content (2012) employed a design similar to that of Hurewitz et al. (2006) and suggested that only discrete properties are processed automatically. In these studies, as in others in the field, there was an effort to assure that continuous properties would not interfere. However, there are almost no reports on efforts to check that continuous properties indeed did not interfere. For example, Odic et al. (2013) presented participants (children and adults) two arrays of dots and asked which array contained more dots. In 50% of the trials, the array with more dots had larger cumulative area (congruent trials), while the rest of the trials were incongruent. The authors found no congruity effect of cumulative area and concluded that participants did not rely on continuous properties to make their decisions. This holds true for cumulative area but not necessarily for other continuous properties that were not investigated. In contrast to those results, Gebuis and Reynvoet (2012b) compared brain activity while participants passively viewed dot arrays or had to actively compare their numerosities. The authors found that numerosity was extracted only when it was relevant to the task, while continuous properties were always extracted. In a psychophysical study, Burr and Ross (2008) suggested that number, similarly to other basic visual properties such as color, contrast, and size, is an adaptable visual property. Hence, numerosity is a basic visual property. However, based on that work, Durgin (2008) suggested that Burr and Ross (2008) actually adapted participants to density.

As demonstrated by the studies mentioned above, findings regarding the role of discrete and continuous properties in estimation and discrimination of magnitudes are inconsistent and often contradicting. Some studies suggest that discrete properties are processed automatically (i.e., Piazza et al., 2004, 2007; Barth, 2008; Burr and Ross, 2008; Cantlon et al., 2009; Piazza, 2010; Soltész et al., 2010), or that *only* discrete properties are being processed automatically (e.g., Nys and Content, 2012). Other studies suggest the opposite; namely, that continuous magnitudes are processed automatically (Clearfield and Mix, 1999, developmental study); (Gebuis and Reynvoet, 2012a,b), and that numerosities affect performance only if relevant to the task (Gebuis and Reynvoet, 2012b). There are also findings proposing that both dimensions are processed automatically, but with different levels of automaticity (i.e., Hurewitz et al., 2006). Consequently, some studies tried to directly investigate the source of the inconsistencies in the literature. Gebuis and Reynvoet (2012a) controlled continuous properties differently in four comparative judgment tasks and reported different result patterns for each task. Specifically, the influence of the continuous properties increased with the number of continuous cues available for the participants. Thus, variability in the degree of available continuous properties might be one of the sources for the contradicting reports in the literature. Tokita and Ishiguchi (2010) found that in estimation tasks, the total area of the dots in an array affected numerical estimation but only for arrays containing more than five dots. When the same experiment was preceded by practice sessions with feedback, the effect of the continuous properties vanished. Hence, both practice and the numerosity range interacted with the influence of continuous properties. Other works (e.g., Nieder et al., 2006; Tokita and Ishiguchi, 2010) presented successive visual stimuli (e.g., dots) to avoid confounds of continuous properties. This type of presentation confounds numerosity with duration and rhythm; it takes longer to present more stimuli. Equating the time means different rates of presentation, etc. In addition, similar to the way we accumulate numerosity, we can also accumulate surface area. Moreover, when dots are presented in the same location [like in the works of Nieder et al. (2006), and Tokita and Ishiguchi (2010), there is a possibility of a confound from visual aftereffects. Nieder et al. recorded neural activity from the intra parietal sulcus (IPS) of monkeys performing a comparison task, and reported that duration and rhythm affected the activity of 8 out of 58 neurons. They concluded that time and rhythm did not affect performance. However, other brain regions besides the IPS were never tested. It is possible that other parts of the brain are involved in processing of such properties. It is also important to note that Nieder et al.'s work dealt only with quantities of 1–4 (the subitizing range) and that most of the presentation forms were canonical. Hence, it is not clear if the results can be generalized to other quantities. There is also no mention of an attempt to check for the influence of continuous properties in consecutive presentation format. Tokita and Ishiguchi (2012) did not report any attempt to investigate possible contributions of their results to temporal or continuous properties.

Some authors attempted to use other dimensions (e.g., audition) to evade the above mentioned problems. However, it seems that other dimensions are susceptible to similar difficulties. For example, Beran (2012) had monkeys choose between sets of food items, only by hearing the items fall into a container. However, the duration of dropping four items and two items is different, and equalizing the duration means increasing the rhythm. Thus, even in the auditory modality, one encounters the same issues as with visually presented quantities. For a more extensive review of auditory stimuli in developmental studies see Mix et al. (2002).

To summarize, continuous and discrete properties are highly correlated. Consequently, it is hard to exclude the influence of continuous properties and study the influence of a discrete property—numerosity—in isolation. Studies used various methods to control the effects of continuous properties. Those studies worked under the assumption that the experimental design prevented participants from using continuous properties. However, in most of those studies, this assumption was not verified. There was either no attempt to test for possible influence of continuous magnitudes, or, like in Odic et al.'s (2013) study, only the influence of the manipulated continuous property was investigated. Thus, in all the studies mentioned above, it is impossible to be sure that the participants' response was based only on numerosity.

## Alternative suggestion

Relying on continuous magnitudes enables “quick and dirty” estimations; for example, where there are more predators, where there is more food, etc. From the ATOM point of view, the faster we make magnitude estimations, the faster we will be able to plan and execute actions. From an evolutionary perspective, it is sufficient in most cases to make “quick and dirty” estimations. Hence, it makes sense to theorize that if we are born with the ability to discriminate magnitudes, it is due to discriminating continuous and not discrete properties.

Von Aster and Shalev (2007) suggested a four-step developmental model of numerical cognition. The first step in this model occurs in infancy. This step postulated an inherited “core system representation of cardinal magnitude and accompanying functions, such as subitizing, approximation and comparison” (p. 870). The authors' suggestion was based on studies discussed above (Section Methodological issues). In light of the criticism of these studies, and new findings from the last few years (Gebuis and Gevers, 2011; Gebuis and Reynvoet, 2012a,b; Henik et al., 2012), we suggest that earlier abilities have to develop before the first step in the model of Von Aster and Shalev (2007) can occur. Specifically, we consider the ability to estimate continuous properties as the first step in a process that will allow us, later in development, to understand and represent numerosities and symbolic numbers.

**Figure 2:**
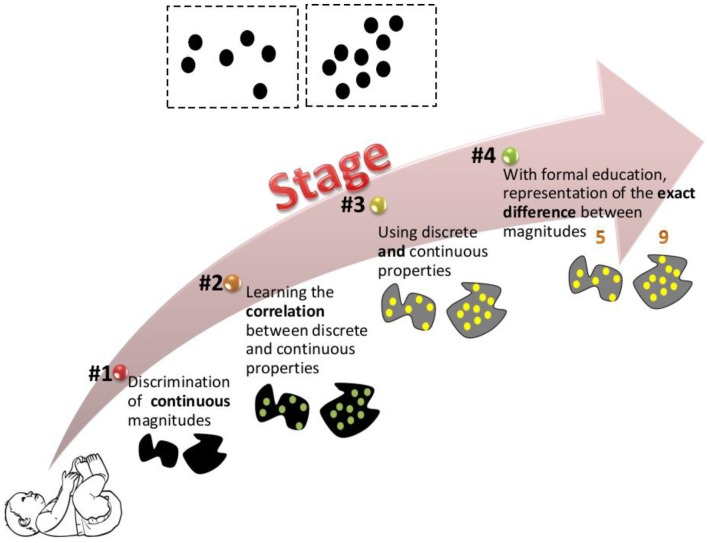
**Suggested developmental model.** The figure illustrates how the two dot arrays at the top of the image are represented at different developmental stages. The model postulates that we are born with the ability to discriminate between continuous properties. During development we learn the correlation between discrete and continuous properties. This allows us to use both discrete and continuous properties to estimate magnitudes. Later, with formal education we are able to represent the exact difference between different magnitudes.

Our suggested model is illustrated in Figure 2. This model presents the ways in which magnitudes are represented and discriminated at different developmental stages. In line with other theories, we postulate an innate ability to discriminate magnitudes. However, we suggest that the initial ability is restricted to continuous magnitudes (stage 1 in our model). Two reasons lead to this suggestion. First, it has been suggested that when appropriate control is exerted, continuous rather than discrete properties seem to dominate infant performance (Clearfield and Mix, 1999; Gebuis and Gevers, 2011; Gebuis and Reynvoet, 2012a,b). Second, most of the magnitude discrimination studies in newborns and infants were investigated using the visual domain, and as such, may be limited by visual acuity of the newborn, which is about 12–25 times worse than that of an adult (Dobson and Teller, 1978; Banks and Shannon, 2013). With age, discrimination between magnitudes improves (Brannon et al., 2006). During development, we are constantly exposed to the environmental correlation between discrete and continuous properties. Thus, in stage 2 we begin to notice this correlation: larger area and density usually mean more numerous. Piaget's (1952) report that children judged five widely spaced coins to be more numerous than five densely spaced coins is an example of overgeneralization of that correlation. In stage 3, children are able to use both discrete and continuous properties to discriminate magnitudes. With formal education, enumeration skills are developed and a child might know not only that array “a” is more numerous than array “b,” but also by how much (stage 4). Further developmental studies are needed to empirically test the suggested model and to specify the age ranges in which the different stages take place.

## Conclusion

We reviewed studies investigating comparative judgment of dot arrays and showed the contradicting results they have found. We suggest that these conflicting results are due to the fact that it is physically impossible to completely neutralize the effect of continuous properties, not only in the visual domain. The different ways that different studies chose to deal with this confound has led to variability in the results and the conclusions. Thus, in our opinion, theories such as the number sense and the ANS, which consider numerosity to be the most salient cue and the basis for our numerical abilities, should be revised to include continuous properties. We further suggest that directly studying the contribution of continuous properties to the ability to discriminate magnitudes, throughout normal and impaired development, in adults as well as in children, might shed light on the basis of numerical cognition.

### Conflict of interest statement

The authors declare that the research was conducted in the absence of any commercial or financial relationships that could be construed as a potential conflict of interest.
